# Fasting blood glucose-to-glycated hemoglobin ratio for evaluating clinical outcomes in patients with ischemic stroke

**DOI:** 10.3389/fneur.2023.1142084

**Published:** 2023-03-20

**Authors:** Tengfei Shao, Hui Liu, Guochao Yang, Huan Wang, Di Li, Huanyu Ni, Yun Xu, Jinping Zhang

**Affiliations:** ^1^Department of Pharmacy, Affiliated Drum Tower Hospital, Nanjing University Medical School, Nanjing, Jiangsu, China; ^2^China Pharmaceutical University Nanjing Drum Tower Hospital, Nanjing, Jiangsu, China; ^3^Ministry of Education (MOE) Key Laboratory of Model Animal for Disease Study, Model Animal Research Center, Jiangsu Key Laboratory of Molecular Medicine, Medical School, Nanjing University, Nanjing, Jiangsu, China; ^4^Department of Pharmacy, Wuhan Fourth Hospital, Wuhan, Hubei, China; ^5^Department of Pharmacy, Wuhan No. 1 Hospital, Wuhan, Hubei, China; ^6^Department of Neurology, Affiliated Drum Tower Hospital, Nanjing University Medical School, Nanjing, Jiangsu, China

**Keywords:** stress hyperglycemia, acute ischemic stroke, clinical outcomes, fasting blood glucose, glycated hemoglobin

## Abstract

**Background:**

Stress hyperglycemia frequently occurs in patients with acute ischemic stroke (AIS). The influence of stress hyperglycemia on the outcomes of patients with AIS remains ambiguous.

**Methods:**

Data from our institution on patients with AIS between June 2020 and June 2021 were retrospectively analyzed. The severity of the stroke was assessed using the National Institutes of Health Stroke Scale (NIHSS) at admission, and the primary endpoint was functional outcomes. Stress hyperglycemia was measured by the glucose-to-HbA1c ratio. In the multivariable analysis, two models that retained or excluded the NIHSS were adopted to explore the relationship between stress hyperglycemia and outcomes. The receiver operating characteristic curve (ROC) was calculated to determine an optimized cutoff value.

**Results:**

The optimal cutoff value was 1.135. When all patients were included, model 1 did not find an association between the glucose-to-HbA1c ratio and functional outcomes. In model 2, the glucose-to-HbA1c ratio^×10^ (Glucose-to-HbA1c ratio ×10) was the independent predictor of functional outcomes (OR 1.19, 95% CI 1.07–1.33, *p* < 0.01). Separately, in patients without diabetes, the glucose-to-HbA1c ratio^×10^ was the independent predictor of functional outcomes in both model 1 (OR 1.37, 95% CI 1.08–1.73, *p* = 0.01) and model 2 (OR 1.48, 95% CI 1.22–1.79, *p* < 0.01), but not in patients with diabetes. In addition, the glucose-to-HbA1c ratio^×10^ was the independent predictor of stroke severity (OR 1.16, 95% CI 1.05–1.28, *p* < 0.01).

**Conclusion:**

The glucose-to-HbA1c ratio was associated with more severe AIS. Specifically, the glucose-to-HbA1c ratio was associated with the functional outcomes in patients without diabetes but not in patients with diabetes.

## Introduction

Stress hyperglycemia is regarded as transient hyperglycemia secondary to inflammatory and neurohormonal disturbances in the context of acute illnesses (1–3). To our knowledge, the relationship between stress hyperglycemia and clinical outcomes has been studied in patients with AIS (4) and those with cardiovascular disease (5). In addition, studies have shown that stress hyperglycemia in patients with myocardial infarction is associated with an increased risk of in-hospital mortality, whether or not patients have diabetes (5). Stress hyperglycemia is frequently observed in patients with AIS (6), whether or not they have diabetes. Previous studies focused on patients without diabetes (7) or only considered fasting blood glucose (FBG) (8). Therefore, the relationship between stress hyperglycemia and clinical outcomes after AIS in patients with or without diabetes has not been well characterized.

Recently, an increasing number of studies have focused on the role of background blood glucose in assessing stress hyperglycemia. According to Roberts et al. (1), the stress hyperglycemia ratio (SHR), a novel index, can be used for accessing stress hyperglycemia. It was defined as admission blood glucose divided by the estimated mean blood glucose derived from glycated hemoglobin (HbA1c). Furthermore, considering HbA1c is a well-validated index that reflects the background blood glucose levels over the past 8–12 weeks (9) and that FBG is a more recognized index of the current blood glucose level, several studies have begun to use the FBG/HbA1c ratio to assess relative hyperglycemia, which is calculated by dividing FBG by HbA1c (7, 10, 11). Therefore, this calculation formula is convenient, practical, and reasonable.

Using this easy-to-perform method to identify and quantify stress hyperglycemia, our study explored the relationship between stress hyperglycemia and clinical outcomes in patients with AIS.

## Methods

### Study participants

A total of 283 patients with a clinical diagnosis of AIS derived from our institution between June 2020 and June 2021 were finally enrolled. AIS was diagnosed according to the World Health Organization criteria (12) and confirmed by brain computerized tomography (CT) or magnetic resonance imaging (MRI). The severity of the stroke was assessed using the National Institutes of Health Stroke Scale (NIHSS) (13) by trained neurologists at admission. The severity of the stroke was classified as mild stroke (NIHSS score ≤4 at admission) and moderate-to-severe stroke (NIHSS score>4 at admission). Stroke types were classified as large-artery atherosclerosis, cardioembolic, small vessel disease, and others or undetermined.

Patients were eligible for the study if they were >18 years old, were admitted within 7 days after the occurrence of the stroke, underwent routine laboratory investigations after an overnight fast on the first day after admission, underwent MRI or CT, and had the diagnosis of AIS confirmed after admission. Patients were excluded from the study if they had incomplete clinical data or a premorbid mRS score of >1.

### Data collection

The clinical data and baseline demographics of patients were consecutively collected through an electronic medical record system. All enrolled patients received routine therapy and nursing care according to their conditions.

### Assessment of stress hyperglycemia

Stress hyperglycemia was evaluated by the glucose-to-HbA1c ratio. We used the following formula to calculate the glucose-to-HbA1c ratio: FPG (mmol/L)/HbA1c (%). This index reflected the extent of increase in acute blood glucose level based on the background blood glucose level.

### Follow-up and outcomes

All patients completed a 12-month follow-up. During the follow-up period, outcomes were recorded using our hospital's electronic medical record system or through a telephone interview. Functional outcomes were measured using the mRS score at 1 year. A score of 3–6 was defined as a poor functional outcome. Patients' stroke recurrence and all-cause death were also recorded as clinical outcomes.

### Statistical analysis

Independent sample *t*-tests or the Mann-Whitney U-test were used for continuous variables, and the chi-squared test or Fisher's exact test was used for binary variables to perform univariable analyses as appropriate. The receiver operating characteristic curve (ROC) was used to determine an optimized cutoff value for the glucose-to-HbA1c ratio in predicting poor functional outcomes. According to the optimized cutoff, the characteristics of patients with high and low glucose-to-HbA1c ratios were compared.

Univariable analysis variables with significant effects were included in the multivariable regression analysis for further analysis to identify independent predictors of poor functional outcome, stroke recurrence, and stroke severity. To explore whether stress hyperglycemia was related to stroke severity, the two models that retained or excluded the NIHSS score in the multivariable analysis were adopted. The patients were also divided into three groups: those with diabetes, those without diabetes, and all patients. All results were reported using 95% CIs. A *p*-value of < 0.05 (two-sided) was considered statistically significant. All data were analyzed using the statistical package SPSS (version 23.0; SPSS, Chicago, IL, USA). The study protocol was compliant with the Declaration of Helsinki and was approved by the ethical committee of our institution; individual informed consent was not required. The study was registered in the Chinese Clinical Trial Register (ChiCTR-ROC-17012225).

## Results

### Participant characteristics

**Figure 1:**
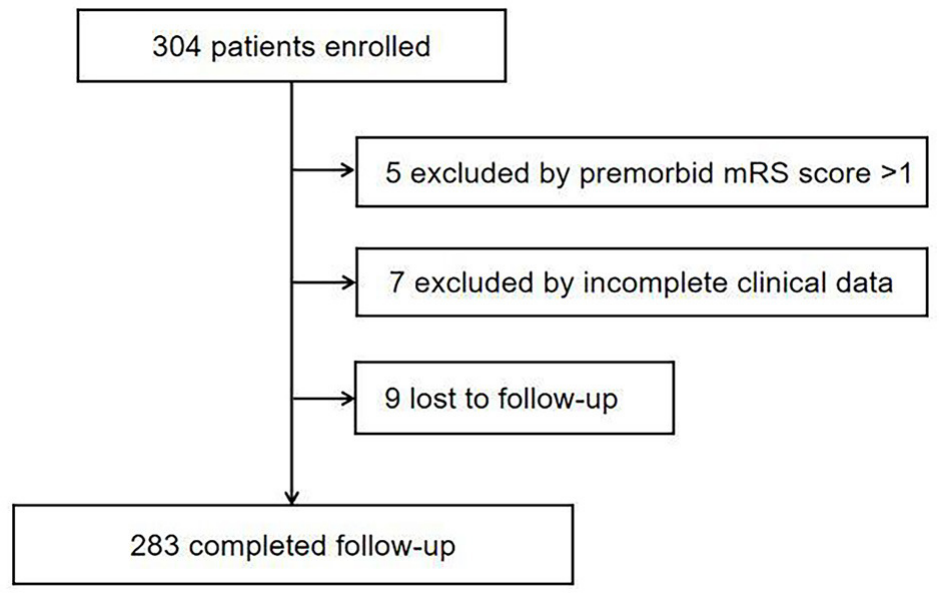
The flowchart of the study.

A total of 283 patients with AIS were finally included in our study, with a median age of 65 years, and 196 (69.2%) of them were men. The baseline demographic and disease characteristics of participants are shown in Table 1. The flowchart of the study is displayed in Figure 1.

**Table 1:** Comparison of low-stress hyperglycemia ratio (glucose-to-HbA1c ratio <1.135) vs. high-stress hyperglycemia ratio (glucose-to-HbA1c ≥ 1.135) in patients with acute ischemic stroke.

	**ALL (*N* = 283)**	**Low Glucose-to-HbA1c ratio (*N* = 180)**	**High Glucose-to-HbA1c ratio (*N* = 103)**	***P* ^#^**
**Baseline characteristics**				
Age, years	65 (57, 73)	64.9 ± 12.1	63.7 ± 13.3	0.44
Gender (men, *n*%)	196 (69.2)	131 (72.8)	65 (63.1)	0.09
BMI, kg/m^2^, median (IQR)	24.5 (23.0, 26.4)	24.5 (23.0, 26.4)	24.7 (22.6, 26.0)	0.75
NIHSS score at admission, median (IQR)	2 (1, 5)	2 (1, 4)	3 (1, 10)	0.02^*^
Mild stroke (NIHSS score ≤4, *n*%)	200 (70.6)	136 (75.6)	64 (62.1)	0.02^*^
**Previous history**, ***n*** **(%)**				
History of stroke	63 (22.2)	43 (23.9)	20 (19.4)	0.38
Coronary heart disease	34 (12.0)	22 (12.2)	12 (11.7)	0.89
Atrial Fibrillation	21 (7.4)	15 (8.3)	6 (5.8)	0.44
Hypertension	197 (69.6)	125 (69.4)	72 (69.9)	0.94
Diabetes	104 (36.7)	64 (35.8)	40 (38.8)	0.61
Smoking	108 (38.1)	73 (40.6)	35 (34.0)	0.27
**Previous drugs**, ***n*** **(%)**				
Antihypertensive agents	158 (55.8)	102 (56.7)	56 (54.4)	0.71
Antidiabetic agents	91 (32.1)	52 (28.9)	39 (37.9)	0.12
Statins	55 (19.4)	44 (24.4)	11 (10.7)	<0.01^*^
Antiplatelets	68 (24.0)	49 (27.2)	19 (18.4)	0.10
**Stroke etiology**, ***n*** **(%)**				0.60^a^
Large-artery atherosclerosis	251 (88.6)	161 (89.4)	90 (87.4)	
Cardioembolic	13 (4.5)	9 (5.0)	4 (3.9)	
Small vessel disease	18 (6.4)	9 (5.0)	9 (8.7)	
Other or undetermined	1 (0.004)	1 (0.005)	0 (0)	
**Recanalization therapy**	34 (12.0)	20 (11.1)	14 (13.6)	0.54
**Hemorrhagic transformation**	9 (3.2)	5 (2.8)	4 (3.9)	0.73^a^
**Biochemical indexes**				
SBP (mmHg), median (IQR)	148 (133, 161)	145.5 (133, 157)	151 (136, 165)	0.07
DBP (mmHg), median (IQR)	81 (75, 92)	81.5 (76, 92)	81 (73, 91)	0.56
FBG (mmol/L), median (IQR)	6.6 (5.6, 8.6)	5.9 (5.3, 6.6)	8.6 (7.3, 10.8)	<0.01^*^
HbA1c (%), median (IQR)	6.0 (5.6, 7.7)	6 (5.6, 7.5)	6.1 (5.6, 7.8)	0.66
Glucose-to-HbA1c ratio, median (IQR)	1.1 (0.9, 1.2)	-	-	-
LDL-C, mg/dl, median (IQR)	2.2 (1.72.9)	2.3 (1.7, 2.9)	2.2 (1.7, 2.8)	0.42
HDL-C, mg/dl, median (IQR)	1.0 (0.8, 1.2)	0.9 (0.8, 1.1)	1.0 (0.8, 1.2)	0.78
TC, mg/dl, median (IQR)	3.8 (3.2, 4.6)	3.9 (3.2, 4.6)	3.8 (3.2, 4.6)	0.61
TG, mg/dl, median (IQR)	1.3 (1.0, 1.8)	1.3 (1.0, 1.8)	1.2 (0.9, 1.8)	0.43
**Outcomes**, ***n*** **(%)**				
mRS score at 1 year	1 (0, 3)	1 (0, 2)	2 (0, 4)	0.03^*^
Poor functional outcome (mRS score>2 at 1 year)	90 (31.8)	44 (24.4)	46 (44.7)	<0.01^*^
Stroke recurrence	30 (10.6)	21 (11.7)	9 (8.7)	0.44
All-cause death at 1 year	10 (23.5)	3 (1.7)	7 (6.8)	0.03^a^

IQR, interquartile range; BMI, body mass index; SBP, systolic blood pressure; DBP, diastolic blood pressure; FBG, fasting blood glucose; HbA1c, glycosylated hemoglobin; LDL-C, low-density lipoprotein cholesterol; HDL-C, high-density lipoprotein cholesterol; TC, total cholesterol; TG, triglyceride; NIHSS, National Institutes of Health Stroke Scale.

^#^Comparison of the low glucose/HbA1c ratio with high glucose/HbA1c ratio.

^*a*^The comparisons were accomplished by Fisher's exact test.

^*^p < 0.05.

### Characteristics of the patients according to the glucose-to-HbA1c ratio

**Figure 2:**
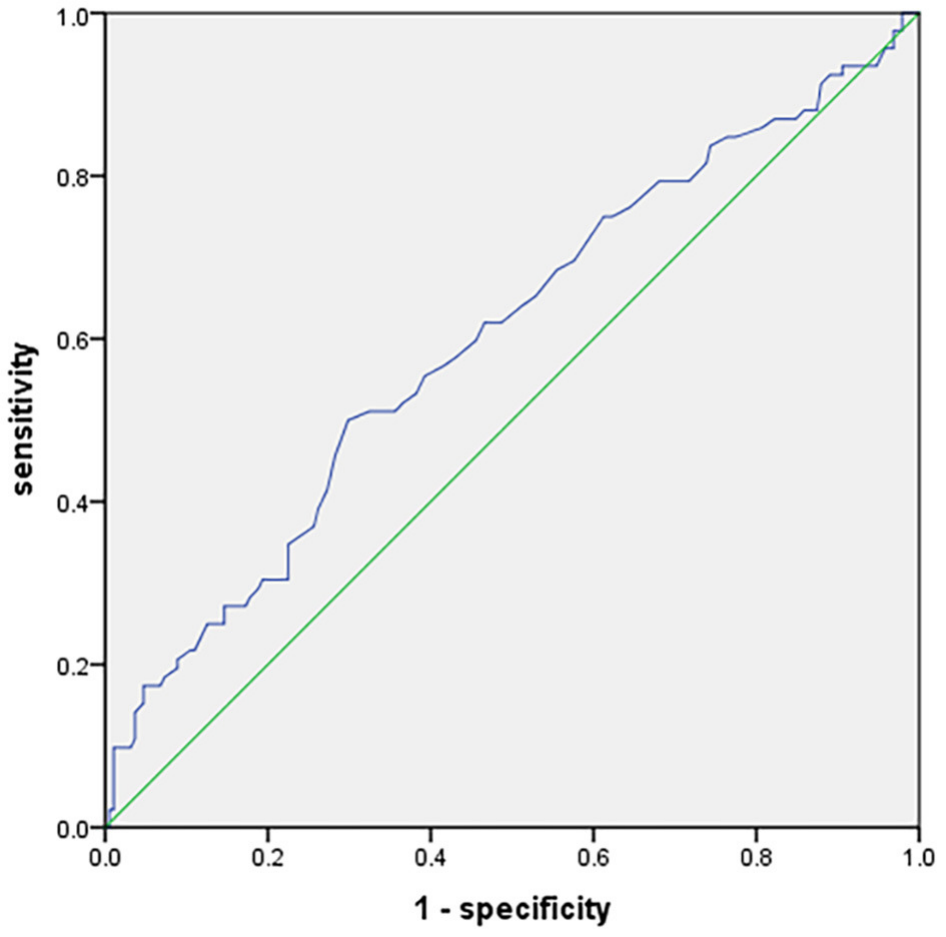
Receiver operating characteristic curve showing optimized cutoff for the glucose-to-HbA1c ratio in predicting poor functional outcomes (mRS score 3–6).

The ROC curve analysis was employed to determine the predicted value of the glucose-to-HbA1c ratio. The optimal cutoff derived from the glucose-to-HbA1c ratio was 1.135, which helped to predict poor functional outcomes in patients with AIS (area under the curve 0.601, 95% CI 0.53–0.67, *p* < 0.01), with 50% sensitivity and 70.2% specificity (Figure 2).

Patients with a higher glucose-to-HbA1c ratio tended to have higher NIHSS scores at admission [3 (1–10) vs. 2 (1–4), *p* = 0.02]. Furthermore, patients with a higher glucose-to-HbA1c ratio were related to an increased risk of poor functional outcomes and mortality at the end of 12 months of follow-up [44.7 vs. 24.4%, *p* < 0.01, 6.8 vs. 1.7%, *p* = 0.03, respectively). However, there was no significant difference in stroke recurrence between the high and low glucose-to-HbA1c ratio groups [8.7 vs. 11.7%, *p* = 0.44] (Table 1).

We did not observe any significant difference regarding stroke etiology between the two groups (*p* = 0.55). In terms of previous drugs, patients in the low glucose-to-HbA1c ratio group had a history of a higher statin usage rate [24.4 vs. 10.7%, *p* < 0.01] (Table 1).

### The associations between glucose-to-HbA1c ratio and poor functional outcomes in patients with or without diabetes

A total of 90 patients had poor functional outcomes at 12 months, including 34 in the diabetes group and 56 in the patients without diabetes (Supplementary Table 1). When all patients were included, the multivariable logistic regression analysis (model 1) found that independent predictors of poor functional outcomes were age (OR 1.05, 95% CI 1.01–1.08, *p* = 0.02), NIHSS score at admission (OR 1.30, 95% CI 1.19–1.42, *p* < 0.01), atrial fibrillation (OR 4.10, 95% CI 1.18–14.28, *p* = 0.03), SBP (OR 1.03, 95% CI 1.01–1.05, *p* < 0.01), and DBP (OR 0.96, 95% CI 0.93–0.99, *p* = 0.02). When the NIHSS score at admission was removed from the multivariable model (model 2), independent predictors of poor functional outcomes were atrial fibrillation (OR 4.49, 95% CI 1.43–14.14, *p* = 0.01), SBP (OR 1.03, 95% CI 1.01–1.05, *p* < 0.01), DBP (OR 0.95, 95% CI 0.92–0.98, *p* < 0.01), and glucose-to-HbA1c ratio^×10^ (OR 1.19, 95% CI 1.07–1.33, *p* < 0.01) (Table 2).

**Table 2:** Independent predictors of poor functional outcomes after 1 year in binary logistic regression analysis.

	**Variables**	**OR**	**95%CI**	***P***
All	**Model 1** ^α^			
	Age	1.05	1.01~1.08	0.02^*^
	NIHSS score at admission	1.30	1.19~1.42	<0.01^*^
	Atrial fibrillation	4.10	1.18~14.28	0.03^*^
	SBP	1.03	1.01~1.05	<0.01^*^
	DBP	0.96	0.93~0.99	0.02^*^
	**Model 2** ^α^			
	Atrial fibrillation	4.49	1.43~14.14	0.01^*^
	SBP	1.03	1.01~1.05	<0.01^*^
	DBP	0.95	0.92~0.98	<0.01^*^
	Glucose-to-HbA1c ratio^×10^	1.19	1.07~1.33	<0.01^*^
With diabetes	**Model 1** ^β^			
	Age	1.06	1.01~1.12	0.03^*^
	NIHSS score at admission	1.23	1.07~1.43	<0.01^*^
	**Model 2** ^β^			
	Age	1.06	1.01~1.11	0.04^*^
	Hypertension	3.41	1.01~11.50	<0.05^*^
Without diabetes	**Model 1** ^γ^			
	Age	1.08	1.03–1.13	<0.01^*^
	Atrial fibrillation	5.17	1.05~25.51	0.04^*^
	NIHSS score at admission	1.28	1.15~1.42	<0.01^*^
	Glucose-to-HbA1c ratio^×10^	1.37	1.08~1.73	0.01^*^
	**Model 2** ^γ^			
	Age	1.06	1.03~1.10	<0.01^*^
	BMI	0.85	0.74~0.98	0.02^*^
	Atrial fibrillation	4.97	1.10~22.39	0.04^*^
	Glucose-to-HbA1c ratio^×10^	1.48	1.22~1.79	<0.01^*^

Glucose-to-HbA1c ratio^×10^= Glucose-to-HbA1c ratio × 10.

Model 1^α^: adjusted for gender, age, BMI, NIHSS score at admission, medical histories of stroke, atrial fibrillation, hypertension, smoking, previous use of statins, antihypertensive agents, SBP, DBP, HDL-C, TG, and glucose-to-HbA1c ratio^×10^.

Model 2^α^: adjusted for gender, age, BMI, medical histories of stroke, atrial fibrillation, hypertension, smoking, previous use of statins and antihypertensive agents, SBP, DBP, HDL-C, TG, and glucose-to-HbA1c ratio^×10^.

Model 1^β^: adjusted for age, NIHSS score at admission, hypertension, DBP, and HbA1c.

Model 2^β^: adjusted for age, hypertension, DBP, and HbA1c.

Model 1^γ^: adjusted for gender, age, BMI, medical history of atrial fibrillation, smoking, NIHSS score at admission, FBG, HDL-C, TG, and glucose-to-HbA1c ratio^×10^.

Model 2^γ^: adjusted for gender, age, BMI, medical history of atrial fibrillation, smoking, FBG, HDL-C, TG, and glucose-to-HbA1c ratio^×10^.

OR, odds ratio; CI, confidence interval; BMI, body mass index; SBP, systolic blood pressure; DBP, diastolic blood pressure; HDL-C, high-density lipoprotein cholesterol; TC, total cholesterol; TG, triglyceride; NIHSS, National Institutes of Health Stroke Scale.

^*^p <0.05.

In patients with diabetes, we did not observe a relationship between the glucose-to-HbA1c ratio and poor functional outcomes. However, in patients without diabetes, the glucose-to-HbA1c ratio^×10^ was the independent predictor in both model 1 (OR 1.37, 95% CI 1.08–1.73, *p* = 0.01) and model 2 (OR 1.48, 95% CI 1.22–1.79, *p* < 0.01) (Table 2).

### The associations between glucose-to-HbA1c ratio and stroke recurrence

In total, 30 patients underwent stroke recurrence during the 12 months of follow-up. There was no difference in the glucose-to-HbA1c ratio between the stroke recurrence and nonrecurrence groups (Supplementary Table 2). In multivariable logistic regression analysis, the glucose-to-HbA1c ratio had no relationship with stroke recurrence (Supplementary Tables 2, 3).

### The associations between glucose-to-HbA1c ratio and stroke severity at admission

The patients were divided into two groups according to the NIHSS score at admission as follows: mild stroke was defined as an NIHSS score ≤4 and moderate-to-severe stroke was defined as an NIHSS score >4. A total of 83 patients had a moderate-to-severe stroke at admission (Supplementary Table 4). The glucose-to-HbA1c ratio^×10^ was related to an increased risk of more severe stroke (OR 1.16, 95% CI 1.05–1.28, *p* < 0.01). In addition, cardioembolic was also associated with a more severe stroke (OR 4.02, 95% CI 1.23–13.21, *p* = 0.02) (Table 3).

**Table 3:** Independent predictors of a moderate-to-severe stroke at admission in binary logistic regression analysis.

**Variables**	**OR**	**95%CI**	***P***
Glucose-to-HbA1c ratio^×10^	1.16	1.05~1.28	<0.01^*^
**Stroke etiology**			
Cardioembolic	4.02	1.23~13.21	0.02^*^
Large-artery atherosclerosis	-	-	-

Glucose-to-HbA1c ratio^×10^= Glucose-to-HbA1c ratio × 10.

OR, odds ratio; CI, confidence interval.

Adjusted for stroke etiology, glucose-to-HbA1c ratio^*^10, high-density lipoprotein cholesterol, and HbA1c.

Moderate-to-severe stroke means an NIHSS score of >4 at admission.

^*^p <0.05.

## Discussion

In this study, we explored the relationship between the glucose-to-HbA1c ratio and the clinical outcomes in patients with AIS. The major findings of the present study were as follows: Stress hyperglycemia, *via* the glucose-to-HbA1c ratio, was related to poor functional outcomes in patients without diabetes but not in patients with diabetes. In addition, regardless of whether patients had diabetes or not, the glucose-to-HbA1c ratio was significantly associated with poor functional outcomes only in model 2, that is, a multivariable analysis excluding NIHSS score at admission. Through analyzing the severity of stroke at admission, we found that the glucose-to-HbA1c ratio was an independent predictor for moderate-to-severe stroke at admission. The association between stress hyperglycemia and poor outcomes tended to be attributed to higher stroke severity in patients with stress hyperglycemia.

Although a range of evidence suggests that stress hyperglycemia is a marker of poor outcomes in patients with AIS (14–21), this relationship is controversial when stroke severity is considered in further analysis (8). However, a recent meta-analysis found that stress hyperglycemia could reflect the extent of ischemic damage and lead to poor clinical outcomes in patients with stroke (22). This finding was consistent with part of our results that stress hyperglycemia is more common in patients with severe stroke, similar to that observed in a previous study (23). However, the underlying mechanism of the relationship between stress hyperglycemia and poor outcomes in patients with AIS is still unclear. In particular, it is necessary to differentiate between patients with and without diabetes.

Our study showed that stress hyperglycemia was a predictor of poor outcomes in patients without diabetes but not in patients with diabetes. Merlino et al. (24) reported that premorbid diabetic status tended to influence outcomes in patients with AIS who were treated with intravenous thrombolysis. Mortality and hemorrhagic complications were significantly increased in patients with more severe stress hyperglycemia only when they were not affected by diabetes. Similar results were also found in several other previous studies (4, 21, 25–27). The underlying mechanism of this phenomenon may be explained by cellular adaptation to hyperglycemia due to physiological readjustments to higher glucose concentrations in patients with diabetes (28, 29). In addition, diabetes may improve antioxidant defenses, which can protect cells from oxidative stress caused by acute hyperglycemia and thus attenuate inflammation caused by oxidative stress (30, 31).

Treatment and risk factor management in patients with AIS greatly influence overall prognosis. Finding indicators that can help us choose different treatments for different patients is therefore crucial, especially in glucose control and intensive glucose-lowering therapy. However, while random blood glucose and FBG are more available and intuitive, both have limitations in ignoring background blood glucose levels and physiological stress responses to AIS. In contrast, using the glucose-to-HbA1c ratio overcomes these shortcomings.

In our study, patients with a higher glucose-to-HbA1c ratio had higher NIHSS scores at admission, which might indicate that stress hyperglycemia was associated with stroke severity, and the glucose-to-HbA1c ratio was an independent predictor of stroke severity. Several previous studies have found that the glucose-to-HbA1c ratio influences clinical outcomes in both patients with and without diabetes (8, 20, 32–34). In these studies, a higher glucose-to-HbA1c ratio was related to poorer outcomes. These results might be explained by poorer outcomes that more severe patients always experience. However, in our study, when the patients were divided into groups with and without diabetes for separate analysis, it was found that the glucose-to-HbA1c ratio did not show a relationship with poor functional outcomes in patients with diabetes, while it was significantly associated with the poor functional outcomes in patients without diabetes.

Regardless, we found that the optimal glucose-to-HbA1c ratio was highly correlated with 1-year functional outcomes and all-cause mortality with the cutoff value of 1.135 but had no relationship with stroke recurrence. Patients with a higher glucose-to-HbA1c ratio had poorer functional outcomes. These results suggested that stress hyperglycemia might influence patients' clinical outcomes. Although the POINT trial found that hyperglycemia was associated with an increased risk of subsequent ischemic stroke (14), there are two possible explanations for the difference. First, the enrolled patients were those who presented with a high-risk TIA or acute minor ischemic stroke, while our study did not limit the stroke characteristics. Second, they defined hyperglycemia as random serum glucose on presentation ≥180 mg/dl, which differs from our study, which used the glucose-to-HbA1c ratio to define hyperglycemia. To our knowledge, diabetes is an independent risk factor for stroke recurrence (35). However, research on the impact of stress hyperglycemia on stroke recurrence is insufficient, and further studies are needed.

Our study had several limitations. First, this was a single-center, retrospective study with a limited sample size, which might reduce the generalizability of the results. Second, since the time of stroke recurrence was not recorded, survival analysis could not be performed, which was a shortcoming of our study. In addition, early control of hyperglycemia might impact the outcomes, as hyperglycemia can exacerbate brain damage in ischemic stroke, which we had not considered. In addition, the time from onset to laboratory investigations might influence our results, which had been ignored. Finally, the absence of 3-month follow-up data was also a shortcoming of this article. Therefore, the impact of stress hyperglycemia on patients with AIS remains a concern, and a multicenter prospective trial with a large sample size is needed in the future. In addition, patients with or without diabetes will also need to be studied separately.

In conclusion, our study showed that the glucose-to-HbA1c ratio was associated with more severe AIS and might be a marker of the severity of the stroke. More specifically, a high glucose-to-HbA1c ratio was associated with poor functional outcomes in patients without diabetes but not in patients with diabetes.

## Data availability statement

The raw data supporting the conclusions of this article will be made available by the authors, without undue reservation.

## Ethics statement

The studies involving human participants were reviewed and approved by Medical Ethics Committee of Affiliated Drum Tower Hospital, Nanjing University Medical School. Written informed consent for participation was not required for this study in accordance with the national legislation and the institutional requirements.

## Author contributions

JZ and YX designed the experiments and revised the manuscript. TS enrolled patients and drafted the manuscript. HL did the analysis work and drafted the manuscript with TS. GY, HW, DL, and HN contributed to patient enrollment and data collection. GY helped revise the manuscript. All authors contributed to the article and approved the submitted version.
