# Supplementary material for: Fasting blood glucose-to-glycated hemoglobin ratio for evaluating clinical outcomes in patients with ischemic stroke
Source: Front Neurol. 2023 Mar 20;14:1142084. doi: 10.3389/fneur.2023.1142084 (PMC10067677; doi:10.3389/fneur.2023.1142084)
Supplement: Supplementary file 1 [file Table_1.docx]

**Supplementary Table 1 Comparison of patients with good functional outcomes with poor functional outcomes**

|  | **All (N=283)** | | | **With Diabetes (N=104)** | | | **Without diabetes (N=179)** | | |
| --- | --- | --- | --- | --- | --- | --- | --- | --- | --- |
|  | mRS 0-2  (N =193) | mRS 3-6  (N =90) | *p* | mRS 0-2  (N =70) | mRS 3-6  (N =34) | *p* | mRS 0-2  (N= 123) | mRS 3-6  (N= 56) | *p* |
| **Baseline Characteristics** | | | | | | | | | |
| Age,years | 63(54~70) | 71(63~80) | <0.01* | 65.2±10.2 | 72.0±10.6 | <0.01* | 59.9±12.3 | 68.9±13.0 | <0.01* |
| Gender (male, n%) | 144(74.6) | 52(57.8) | <0.01* | 50(71.4) | 20(58.8) | 0.20 | 94(76.4) | 32(57.1) | <0.01* |
| BMI, kg/m2, median (IQR) | 24.7(23.3~26.7) | 24.3(22.0~25.4) | 0.03* | 24.6±2.6 | 25.0±3.2 | 0.48 | 24.7(23.4~27.0) | 24.1(22.0~24.8) | <0.01* |
| NIHSS at admission, median (IQR) | 2(1~4) | 5(3~12) | <0.01* | 2(1~4) | 3(2~9) | <0.01* | 2(1~3) | 7.5(3~12) | <0.01* |
| **Previous history, n (%)** | | | | | | | | | |
| History of stroke | 36(18.7) | 27(30.0) | 0.03* | 16(22.9) | 12(35.3) | 0.18 | 20(16.3) | 15(26.8) | 0.10 |
| Coronary heart disease | 20(10.4) | 14(15.6) | 0.21 | 8(11.4) | 9(26.5) | 0.05 | 12(9.8) | 5(8.9) | 0.86 |
| Atrial Fibrillation | 7(3.6) | 14(15.6) | <0.01* | 4(5.7) | 4(11.8) | 0.28 | 3(2.4) | 10(17.9) | <0.01* |
| Hypertension | 127(65.8) | 70(77.8) | 0.04* | 46(65.7) | 30(88.2) | 0.02* | 81(65.9) | 40(71.4) | 0.46 |
| Diabetes | 70(36.5) | 34(37.8) | 0.83 | - | - | - | - | - | - |
| Smoking | 86(44.6) | 22(24.4) | <0.01* | 30(42.9) | 8(23.5) | 0.06 | 56(45.5) | 14(25.0) | <0.01* |
| **Previous drugs, n (%)** | | | | | | | | | |
| Antihypertensive agents | 99(51.3) | 59(65.6) | 0.02* | 39(55.7) | 31(91.2) | <0.01* | 60(48.8) | 28(50.0) | 0.88 |
| Antidiabetic agents | 60(31.1) | 31(34.4) | 0.57 | 54(77.1) | 29(85.3) | 0.33 | - | - | - |
| Statins | 31(16.1) | 24(26.7) | 0.04* | 15(21.4) | 12(35.3) | 0.13 | 16(13.0) | 12(21.4) | 0.15 |
| Antiplatelet | 40(20.7) | 28(31.1) | 0.06 | 17(24.3) | 14(41.2) | 0.08 | 23(18.7) | 14(25.0) | 0.33 |
| **Stroke etiology, n (%)** | | | 0.54^a^ |  |  | 0.34^a^ |  |  | 0.66^a^ |
| Large-artery atherosclerosis | 171(88.6) | 80(89.9) |  | 59(84.3) | 31(91.2) |  | 112(91.1) | 49(87.5) |  |
| Cardioembolic | 7(3.6) | 6(6.7) |  | 3(4.3) | 2(5.9) |  | 4(3.3) | 4(7.1) |  |
| Small vessel disease | 14(7.3) | 4(4.4) |  | 8(11.4) | 1(2.9) |  | 6(4.9) | 3(5.4%) |  |
| Other or undetermined | 1(0.005) | 0(0) |  | 0(0) | 0(0) |  | 1(0.8) | 0(0) |  |
| Recanalization therapy | 20(10.4) | 14(15.6) | 0.21 | 7(10.0) | 2(5.9) | 0.71^a^ | 13(10.6) | 12(21.4) | 0.052 |
| Hemorrhagic transformation | 4(2.1) | 5(5.6) | 0.15^a^ | 2(2.9) | 1(2.9) | 1.00^a^ | 2(1.6) | 4(7.1) | 0.08^a^ |
| **Biochemical indexes** | | | | | | | | | |
| SBP (mmHg), median (IQR) | 145(133~158) | 151(136~166) | 0.04* | 146.4±20.5 | 152.0±17.5 | 0.18 | 146.4±19.3 | 153.2±26.6 | 0.09 |
| DBP (mmHg), median (IQR) | 83(77~92) | 79(72~89) | 0.01* | 83.4±11.2 | 78.2±15.0 | <0.05* | 83(77~95) | 80(75~89) | 0.10 |
| FBG (mmol/L), median (IQR) | 6.3(5.5~8.5) | 7.1(6.0~8.6) | 0.07 | 8.3(6.5~10.6) | 8.2(6.2~10.6) | 0.81 | 5.9(5.3~6.8) | 6.7(5.5~7.9) | 0.02* |
| HbA1c (%), median (IQR) | 6.1(5.6~8.0) | 6.0(5.7~7.2) | 0.48 | 8.2(7.0~9.5) | 7.3(6.4~8.3) | 0.01* | 5.7(5.5~6.1) | 5.8(5.5~6.0) | 0.65 |
| Glucose-to-HbA1c ratio, median (IQR) | 1.0(0.9~1.2) | 1.1(1.0~1.4) | <0.01* | 1.0(0.9~1.3) | 1.0(0.9~1.4) | 0.29 | 1.0(0.9~1.1) | 1.1(1.0~1.4) | <0.01* |
| LDL-C, mg/dl, median (IQR) | 2.3(1.7~2.8) | 2.1(1.6~3.1) | 0.52 | 2.1(1.5~2.6) | 1.9(1.3~2.7) | 0.53 | 2.3(1.9~3.0) | 2.2(1.7~3.2) | 0.84 |
| HDL-C, mg/dl, median (IQR) | 0.9(0.8~1.1) | 1.0(0.9~1.2) | <0.01* | 0.9(0.8~1.0) | 0.9(0.8~1.2) | 0.29 | 1.0(0.8~1.2) | 1.0(0.9~1.2) | 0.02* |
| TC, mg/dl, median (IQR) | 3.8(3.3~4.5) | 3.8(3.1~4.9) | 0.66 | 3.6(3.2~4.2) | 3.4(2.7~4.8) | 0.57 | 4(3.4~4.6) | 3.9(3.3~5.1) | 0.89 |
| TG, mg/dl, median (IQR) | 1.4(1.0~1.9) | 1.1(0.9~1.6) | 0.04* | 1.3(1.0~1.8) | 1.3(0.9~1.7) | 0.65 | 1.4(1.0~2.0) | 1.1(0.9~1.6) | 0.02* |

**Abbreviations:** IQR, interquartile range; BMI, body mass index; SBP, systolic blood pressure; DBP, diastolic blood pressure; FBG, fasting blood glucose; HbA1c, Glycosylated Hemoglobin; LDL-C, low-density lipoprotein cholesterol; HDL-C, high-density lipoprotein cholesterol; TC, total cholesterol; TG, triglyceride. NIHSS, National Institutes of Health Stroke Scale.

# compared the low Glucose/HbA1c ratio with high Glucose/HbA1c ratio;

a The comparisons were accomplished by the Fisher’s exact test;

* p < 0.05

**Supplementary Table 2 Comparing between patients who stroke recurrence during follow-up and those who have no stroke recurrence**

|  | Non-stroke recurrence(N=253) | Stroke recurrence(N=30) | P |
| --- | --- | --- | --- |
| **Baseline Characteristics** | | | |
| Age,years | 65(56~72) | 71(60.3~76.8) | 0.11 |
| Gender (male, n%) | 178(70.4) | 18(60.0) | 0.25 |
| BMI, kg/m2, median (IQR) | 24.7(23.0~26.6) | 24.1(22.8~25.1) | 0.13 |
| NIHSS at admission, median (IQR) | 2(1~5) | 3(0.8~5.5) | 0.58 |
| Mild stroke(NIHSS≤4, n%) | 182(71.9) | 18(60.0) | 0.18 |
| **Previous history, n (%)** | | | |
| History of stroke | 52(20.6) | 11(36.7) | <0.05* |
| Coronary heart disease | 27(10.7) | 7(23.3) | 0.04* |
| Atrial Fibrillation | 15(5.9) | 6(20.0) | <0.001* |
| Hypertension | 173(68.4) | 24(80.0) | 0.19 |
| Diabetes | 90(35.7) | 14(46.7) | 0.24 |
| Smoking | 98(38.7) | 10(33.3) | 0.57 |
| **Previous drugs, n (%)** | | | |
| Antihypertensive agents | 137(54.2) | 21(70.0) | 0.10 |
| Antidiabetic agents | 79(31.2) | 12(40.0) | 0.33 |
| Statins | 47(18.6) | 8(26.7) | 0.29 |
| Antiplatelet | 58(22.9) | 10(33.3) | 0.21 |
| **Stroke etiology, n (%)** | | | 0.74^a^ |
| Large-artery atherosclerosis | 224(88.5) | 27(90.0) |  |
| Cardioembolic | 11(4.3) | 2(6.7) |  |
| Small vessel disease | 17(6.7) | 1(3.3) |  |
| Other or undetermined | 1(0.4) | 0(0) |  |
| Recanalization therapy | 30(11.9) | 4(13.3) | 0.77^a^ |
| Hemorrhagic transformation | 6(2.4) | 3(10.0) | 0.06^a^ |
| **Biochemical indexes** | | | |
| SBP (mmHg), median (IQR) | 148(133.5~161.5) | 146(133~153.25) | 0.38 |
| DBP (mmHg), median (IQR) | 82(75~92) | 80(70.75~90) | 0.13 |
| FBG (mmol/L), median (IQR) | 6.6(5.6~8.55) | 6.6(6.075~8.675) | 0.64 |
| HbA1c (%), median (IQR) | 6.00(5.60~7.70) | 6.30(5.775~8.2) | 0.23 |
| Glucose-to-HbA1c ratio, median (IQR) | 1.05(0.93~1.245) | 1.04(0.94~1.15) | 0.64 |
| LDL-C, mg/dl, median (IQR) | 2.23(1.725~2.86) | 2.155(1.4175~2.89) | 0.34 |
| HDL-C, mg/dl, median (IQR) | 0.95(0.79~1.17) | 0.975(0.83~1.0475) | 0.98 |
| TC, mg/dl, median (IQR) | 3.83(3.265~4.605) | 3.7(3.0075~5.1825) | 0.49 |
| TG, mg/dl, median (IQR) | 1.28(0.97~1.8) | 1.2(0.92~1.955) | 0.75 |

**Abbreviations:** IQR: interquartile range; BMI: body mass index; SBP: systolic blood pressure; DBP: diastolic blood pressure; FBG, fasting blood glucose; HbA1c: Glycosylated Hemoglobin; LDL-C: low-density lipoprotein cholesterol; HDL-C: high-density lipoprotein cholesterol; TC: total cholesterol; TG: triglyceride. NIHSS: National Institutes of Health Stroke Scale.

^a^ The comparisons were accomplished by the Fisher’s exact test;

* p < 0.05.

**Supplementary Table 3**  **Predictors of stroke recurrence in binary logistic regression analysis**

| Variables | OR | 95%CI | P |
| --- | --- | --- | --- |
| Stroke of history | 1.90 | 0.82~4.39 | 0.13 |
| Coronary heart disease | 1.67 | 0.59~4.74 | 0.34 |
| Atrial Fibrillation | 2.97 | 0.96~9.19 | 0.06 |

**Abbreviations:** OR, odds ratio; and CI, confidence interval.

Adjust by history of stroke, coronary heart disease, atrial Fibrillation.

**Supplementary Table 4 Comparing between patients with mild stroke and with moderate to severe stroke.**

|  | Mild stroke  (N=200) | Moderate to severe stroke (N=83) | P |
| --- | --- | --- | --- |
| **Baseline Characteristics** | | | |
| Age,years | 65(56.3~73) | 64(58~71) | 0.66 |
| Gender (male, n%) | 144(72.0) | 52(62.7) | 0.12 |
| BMI, kg/m2, median (IQR) | 24.7(23.0~26.4) | 24.5(22.3~26.0) | 0.39 |
| **Previous history, n (%)** |  |  |  |
| History of stroke | 43(21.5) | 20(24.1) | 0.63 |
| Coronary heart disease | 27(13.5) | 7(8.4) | 0.23 |
| Atrial Fibrillation | 13(6.5) | 8(9.6) | 0.40 |
| Hypertension | 145(72.5) | 52(62.7) | 0.10 |
| Diabetes | 76(38.2) | 28(33.7) | 0.48 |
| Smoking | 78(39.0) | 30(36.1) | 0.65 |
| **Previous drugs, n (%)** |  |  |  |
| Antihypertensive agents | 116(58.0) | 42(50.6) | 0.25 |
| Antidiabetic agents | 67(33.5) | 24(28.9) | 0.45 |
| Statins | 36(18.0) | 19(22.9) | 0.34 |
| Antiplatelet | 46(23.0) | 22(26.5) | 0.53 |
| **Stroke etiology, n (%)** | | | <0.01^a^* |
| Large-artery atherosclerosis | 178(89.0) | 73(88.0) |  |
| Cardioembolic | 5(2.5) | 8(9.6) |  |
| Small vessel disease | 17(8.5) | 1(1.2) |  |
| Other or undetermined | 0 | 1(1.2) |  |
| Recanalization therapy | 7(3.5) | 27(32.5) | <0.01^*^ |
| Hemorrhagic transformation | 5(2.5) | 4(4.8) | 0.46 |
| **Biochemical indexes** | | | |
| SBP (mmHg), median (IQR) | 147(133~160) | 150(135~162) | 0.53 |
| DBP (mmHg), median (IQR) | 82(75~92) | 81(74~91) | 0.30 |
| FBG (mmol/L), median (IQR) | 6.5(5.5~8.6) | 6.9(5.7~8.6) | 0.30 |
| HbA1c (%), median (IQR) | 6.15(5.6~7.975) | 6(5.5~7.2) | <0.05* |
| Glucose-to-HbA1c ratio, median (IQR) | 1.0(0.9~1.2) | 1.1(1.0~1.4) | <0.01* |
| LDL-C, mg/dl, median (IQR) | 2.2(1.7~2.8) | 2.3(1.7~3.1) | 0.43 |
| HDL-C, mg/dl, median (IQR) | 0.9(0.8~1.1) | 1.0(0.8~1.3) | <0.01* |
| TC, mg/dl, median (IQR) | 3.8(3.2~4.5) | 3.9(3.3~5.1) | 0.20 |
| TG, mg/dl, median (IQR) | 1.4(1.0~1.9) | 1.2(1.0~1.6) | 0.18 |

**Abbreviations**: IQR, interquartile range; BMI, body mass index; SBP, systolic blood pressure; DBP, diastolic blood pressure; FBG, fasting blood glucose; HbA1c, Glycosylated Hemoglobin; LDL-C, low-density lipoprotein cholesterol; HDL-C, high-density lipoprotein cholesterol; TC, total cholesterol; TG, triglyceride.

The patients were divided into 2 groups according to the NIHSS score at admission as follows: mild stroke, NIHSS≤4; moderate to severe stroke,NIHSS>4.

^a^ The comparisons were accomplished by the Fisher’s exact test;

* p < 0.05.
